# Effect of MAH-g-PLA on the Properties of Wood Fiber/Polylactic Acid Composites

**DOI:** 10.3390/polym9110591

**Published:** 2017-11-09

**Authors:** Lei Zhang, Shanshan Lv, Ce Sun, Lu Wan, Haiyan Tan, Yanhua Zhang

**Affiliations:** College of Materials Science and Engineering, Key Laboratory of Bio-based Material Science and Technology, Northeast Forestry University, Harbin 150040, China; zhangleilay@nefu.edu.cn (L.Z.); lvshpolymer@163.com (S.L.); sunce@nefu.edu.cn (C.S.); wl1547609092@nefu.edu.cn (L.W.); tanzzx168@163.com (H.T.)

**Keywords:** PLA, wood fiber, maleic anhydride (MAH), melt grafting, interfacial compatibilizer

## Abstract

Maleic anhydride (MAH) was used as the grafting monomer, which was prepared by melt grafting reaction in the twin screw extruder with dicumyl peroxide (DCP) as the initiator, polylactic acid grafted with maleic anhydride (MAH-g-PLA) was successfully prepared as the interface compatibilizer. The PLA/Wood fiber/MAH-g-PLA composites were prepared by melt blending and injection molding with different proportions of compatibilizer added, within which PLA was for the matrix phase and wood fiber was for the reinforcing phase. The crystallinity, microstructure, thermal stability and dynamic thermomechanical property of the composites were studied by X-ray diffraction (XRD), scanning electron microscope (SEM), thermo gravimetric analyzer (TGA) and dynamic mechanical thermal analysis (DMA). Furthermore, the mechanical and water absorption properties of the composites were also characterized. Results showed that the tensile strength and flexural strength of the composites attained the highest at 30% MAH-g-PLA added, where the crystallinity of the composites also showed the highest value. DMA results showed that the addition of MAH-g-PLA interfacial compatibilizer increased the loss modulus of the composites and improved the toughness. Scanning electron microscopy (SEM) showed that when the MAH-g-PLA was used, wood fiber is well dispersed in the PLA matrix phase, and that the interfacial compatibility between the matrix and the enhanced phase was improved. Therefore, the addition of MAH-g-PLA could improve the interfacial compatibility of PLA/Wood fiber composites and improve the mechanical properties of the composites.

## 1. Introduction

With the continuous development of an economic society, people's production and life are increasingly dependent on non-renewable fossil fuels, such as petroleum, and coal, but the environmental pollution caused by using these products is the main issue for their development and utilization [1]. Therefore, finding green materials that can replace petroleum-based products to ease the pressure of resource shortages is in great necessity. In recent years, one strong trend is to explore and develop bio-based composites in academic and industrial application, aiming at not only reducing the influence of fossil energy to natural environment, but also achieving multiple functionalities of green materials. Polylactic acid (PLA) that shows high strength, good processability, excellent mechanical properties is one kind of thermoplastic resin, produced by the polymerization of lactic acid monomer [2,3,4]. Lactic acid can be made from the fermentation of corn, potato, cotton, hemp and other plants [3,5,6,7,8], therefore, the raw materials for PLA are recyclable. Compared to PLA with common resins in industrial products, such as polyolefin, polyethylene terephthalate and other petroleum-based plastic, PLA is biodegradable, belonging to green, non-polluting material [9,10]. However, there are drawbacks in using PLA as a raw material, such as its brittleness, hydrophobicity, low impact strength, high price and poor performance at high temperature and humidity, which significantly limit its wide utilization in industrial applications. Therefore, developing an environmental-friendly approach to overcoming the shortages of PLA and manufacture high-performance PLA-based materials is important.

Due to the good specific strength, modulus, low density, short growth cycle and other characteristics, wood fiber is a good alternative for replacing the current use of glass or aramid fiber [11,12,13,14]. In addition, wood fiber has a wide range of sources, leading to low cost in applications. Therefore, wood fiber is a promising candidate to reinforce PLA materials, not only because it can improve mechanical performances and water-resistance of PLA-based material, but it can also lower manufacturing costs, showing wide application prospects in packaging materials, automotive decorative parts and many other areas [15,16,17]. Meanwhile, the wood fiber can also improve the brittleness of PLA composites, and restrict the corrosion of composites. The preparation process of PLA-based composites reinforced with wood fiber is more complicated than petroleum-based plastic such as polyethylene, mainly due to the unstable properties of PLA during preparation. PLA is easy to break down at high temperature, so preparation temperature needs to be well controlled in the process of preparing composites [18]. Researchers have found that the composite temperature during PLA/Wood fiber preparation has a significant effect on the mechanical properties of the composites [19]. As the molecular weight of PLA may be reduced in the extrusion or injection molding process, the preparation process of composites shows a great impact on the overall performance of the composite materials [20,21,22,23]. The common preparation methods for PLA/Wood fiber composites include melt extrusion, high-speed mixing, physical mixing, etc. [24]. In melt extrusion method, the composite material has the best comprehensive performance when the heating time and shear strength of the production process are moderate [25]. To reinforce PLA with wood fiber, it should be considered that the poor compatibility of wood fiber and PLA can be the main problem affecting the properties of the PLA-based composites. The difficulty in the preparation of the composites therefore is how to improve the interfacial compatibility of the composites and how to achieve better fusion of the molecules at interface [26]. At present, adding glycerol or silicane coupling agents are the common approaches for improving interfacial compatibility. In this paper, we offer a novel modification method for PLA to prepare a new interfacial compatibilizer, which can be added into PLA/Wood fiber composites to achieve effective coupling between the matrix phase and the filling phase.

In this paper, via melt grafting reaction in the twin screw extruder, maleic anhydride grafted polylactic acid (MAH-g-PLA) was prepared by using maleic anhydride (MAH) as grafting monomer and dicumyl peroxide (DCP) as initiator. Different amounts of prepared MAH-g-PLA were used as an interfacial compatibilizer to increase the interfacial compatibility of PLA/Wood fiber composite system, leading to the successful preparation of PLA/Wood fiber/MAH-g-PLA composites. The mechanical properties, thermal stability, crystallinity and interfacial compatibility of the composites were studied, with the aim of investigating the structure–property relationship between MAH-g-PLA content and the performance of PLA/Wood fiber/MAH-g-PLA composites.

## 2. ExperimentalMaterials and Methods

### 2.1. Materials

PLA 4032D, extrusion grade, was purchased from Nature Works, with a weight average molecular weight of 1.72 × 10^6^. The density of PLA is 1.25 g cm^−3^, and the melt point is 165 °C. PLA was pre-dried at 50 °C for 12 h to remove water. Wood fiber (the average aspect ratio is about 9.8, and the length is about 28–30 mm) was obtained from Baiquan wood plastic composite material base (Qiqihar, China). Maleic anhydride (MAH) was provided by Tianjin Fuchen Chemical Reagent Factory (Tianjin, China). Dicumyl peroxide (DCP) used as the initiator of grafting reaction was obtained from Shanghai Wing King Industrial Co., Ltd. (Shanghai, China). Other reagents, analytical grade, were received from Tianjin Yongda Chemical Reagent Development Center (Tianjin, China).

### 2.2. MAH-g-PLA Preparation

PLA particles were placed in a vacuum oven and dried at 50 °C for 12 h. First, the MAH and DCP were accurately weighed according to a certain ratio, and dissolved in acetone. Then, the acetone solution was poured into a beaker containing PLA particles and uniformly dispersed by stirring. The grafting reaction of different raw materials ratios was shown in Table 1. Finally, the obtained mixture was added to a twin screw extruder (SHJ type, Nanjing Jie End Electrical and Mechanical Services Limited, Nanjing, China) and subjected to a melt grafting reaction initiated by the DCP. After grafting, the samples were cooled down and pulverized back. Extruder parameters: temperature (170 °C), screw speed (100 r/min), feed rate (30 r/min).

### 2.3. FT-IR of MAH-g-PLA

The MAH-g-PLA was characterized by a Nicolet 460 Fourier transform infrared spectroscopy (FTIR) spectrophotometer (Shanghai, China). The dried grafted powder and potassium bromide (KBr) (2 mg powder/150 mg KBr) were pressed into a tray for FTIR measurement.

### 2.4. Determination of Grafting Degree

**Scheme 1:**
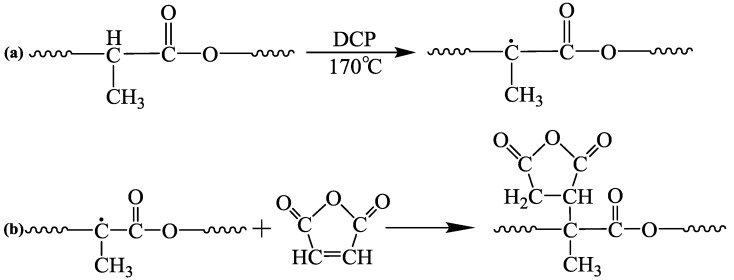
The scheme diagram of polylactic acid (PLA) grafted with maleic anhydride (MAH).

A total of 3 g of the grafted samples prepared above were weighed and placed in a 500 mL four-necked flask. A total of 40 mL tetrahydrofuran was added to the four-necked flask and heated at 50 °C until the sample was completely dissolved. The MAH-g-PLA in the solution was then precipitated with anhydrous ethanol, filtered and washed, and finally placed in a vacuum oven at 50 °C for 12 h. Determination of grafting degree by chemical titration: the purified MAH-g-PLA was weighed into a 100 mL Erlenmeyer flask with the sample weight of 0.4 g, and then 50 mL tetrahydrofuran was added. Two drops of thymol blue/DMF indicator were added dropwise, and the solution was then titrated with 0.05 mol/L potassium hydroxide ethanol solution and with 0.05 mol/L HCl-isopropanol solution. In this research, when the concentration of maleic anhydride was 1.5 g, the grafting degree reached the highest. The Scheme 1 was the chemical reaction of maleic anhydride monomer grafted PLA monomer.

### 2.5. Preparation of PLA/Wood Fiber/MAH-g-PLA Composites

**Figure 1:**
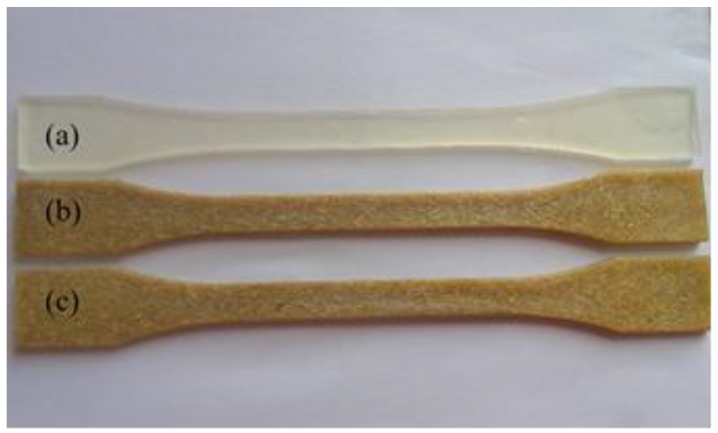
The shape of the composite materials. (a) PLA; (b) PLA/Wood; (c) PLA/Wood fiber/polylactic acid grafted with maleic anhydride (MAH-g-PLA).

PLA, wood fiber and MAH-g-PLA were placed in a vacuum oven at 50 °C for 24 h. A certain ratio of PLA, wood fiber, MAH-g-PLA mixed, mechanical mixing was used to make it evenly mixed. The additional amounts of MAH-g-PLA were 0, 10, 20, 30, 40, or 50 wt %. The amount of PLA and MAH-g-PLA were kept constant, and the mass ratio of wood fiber to (PLA and MAH-g-PLA) was 2:8. The specific amount of different raw materials was shown in Table 2. The obtained mixture was added to the twin screw extruder, and the raw materials in the machine were evenly cut and blended by the screw. The temperature of the screw at five zones were 150, 170, 170, 170 and 135 °C respectively, and the screw speed was 50 rpm. After extruding, the prepared wood–plastic particles were poured into the experimental micro-injection molding machine (Shanghai Xin Shuo Precision Machinery Co., Ltd, Shanghai, China), and the injection molding machine parameters were: injection temperature, 180 °C; injection pressure, 0.6 MPa; heating time, 4 min; packing time, 10 s. The composite material shape of the final injection molding was the dumbbell-shaped spline (as shown in Figure 1).

### 2.6. Characterization of the PLA/Wood Fiber/MAH-g-PLA Composites

#### 2.6.1. X-ray Diffraction and Crystallinity

X-ray diffraction (XRD) data were measured using an X-ray diffraction apparatus (Rigaku D/max 220, Tokyo, Japan). Using a Cu-Kα radiation (λ = 0.1542 nm) as an X-ray source, the generator was set at 32 kV and 30 mA, and the Ni filter was used to extract Kα radiation. The data was collected in the range of a series of scattering angles (2θ) of 5°–40° and the scanning rate was set to 5° min^−1^. All measurements were made at room temperature under atmospheric pressure. According to the Segal method, crystallinity was calculated as Equation (1):

(1) $$\mathrm{CI}(100\%)=(1-\frac{I_{\min}}{I_{\max}})\times 100$$

*I*_max_ represents the maximum intensity of the crystalline region of the material, and *I*_min_ represents the strength of the amorphous region of the material.

#### 2.6.2. Mechanical Properties.

The mechanical properties of the composites were tested on the CMT-5504 universal testing machine (Shenzhen, China SANS testing machine). The tensile strength and elongation at break of the composite were measured according to the ASTM D638-10 method at a crosshead speed of 20 mm/min. The bending strength was measured according to ASTM D790-10 at a crosshead speed of 2 mm/min. The sample geometry is 100 × 10 × 4 mm^3^ (length × width × thickness), each sample was subjected to at least 5 repetitive tests to obtain an average.

#### 2.6.3. Thermal Gravimetric Analysis

Thermal stability of PLA/Wood fiber/MAH-g-PLA composites was measured by thermos gravimetric analysis (TGA). The TGA data was obtained from room temperature to 600 °C at a heating rate of 10 °C/min and an argon flow rate of 50 mL/min on TG 209 F3 (NETZSCH, Shanghai, China).

#### 2.6.4. Dynamic Mechanical Thermal Analysis

Dynamic mechanical thermal analysis (DMA) was performed on TA Instruments (New Castle, DE, USA) RSA3 with three-point bending fixtures. The temperature was raised from 30 °C to 110 °C at a heating rate of 1.5 °C/min and a frequency of 1 Hz in the automatic tension mode (0.02% deformation). The sample size is 50 × 10 × 4 mm^3^ (length × width × thickness), using a span of 40 mm.

#### 2.6.5. Water Absorption

The water absorption of the composite was measured according to ASTM D570-98 (Reapproved 2005). The samples were placed in a vacuum oven at 50 °C for 12 h, weighed with an electronic balance (*W*_0_), and were then immersed in deionized water for 12 days until the material reached the water saturation state. The samples were taken on different days and the water was removed from the surface of the material with absorbent paper, weighed (*W_n_*), *n* = 1, 2, 3,… The initial size of the samples was 30 × 10 × 4 mm^3^ (length × width × thickness), and each sample measured at least 5 averages. The water absorption of the composites was calculated as Equation (2):

(2) $$\mathrm{Water}\;\mathrm{Absorption}=\frac{W_{n}-W_{0}}{W_{0}}\times 100\%$$

#### 2.6.6. Scanning Electron Microscopy

The cross-sectional morphology of PLA/Wood fiber/MAH-g-PLA composites was characterized by scanning electron microscopy (SEM) (QUANTA 200, FEI, Shanghai, China). The SEM was operated at an accelerating voltage of 10 kV. The sample was cooled in liquid nitrogen to produce brittle failure and a flat fracture surface was obtained. Before the SEM observation, the fracture surface was coated with gold sputtering.

## 3. Result

### 3.1. FTIR Spectra of MAH-g-PLA

**Figure 2:**
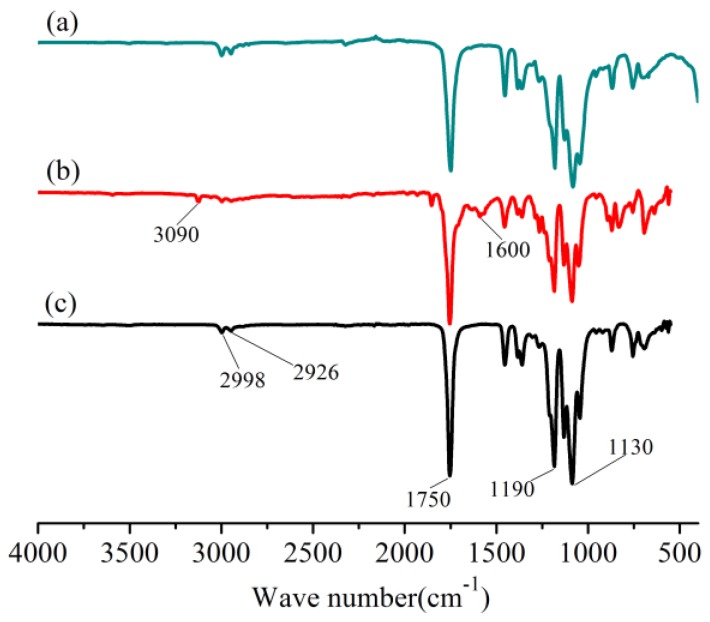
Fourier transform infrared (FTIR) spectra of grafting reaction. (a) PLA; (b) MAH; (c) MAH-g-PLA.

The MAH-g-PLA monomer of the highest grafting degree was characterized by FTIR. Figure 2 showed the FTIR spectrum of pristine PLA, MAH, MAH-g-PLA. From the infrared spectrum of pristine PLA (shown in Figure 2(a)), it can be seen that the asymmetric stretching vibration peak of C–H was at 2998 cm^−1^; a strong characteristic absorption peak of C=O group was at 1750 cm^−1^; the stretching vibration peaks of C–O–C in PLA were at 1190 cm^−1^ and 1130 cm^−1^. From the infrared absorption spectrum of MAH (shown in Figure 2(b)), it can be observed that the out-of-plane bending vibration peak of alkene of =C–H at 3090 cm^−1^ was found compared with the infrared spectrum of MAH-g-PLA (Figure 2(c)), and the telescopic vibration peak of –C=C was at 1600 cm^−1^. As can also be seen from Figure 2(c), the C–O–C absorption peaks were at 1190 cm^−1^, 1130 cm^−1^ and 1093 cm^−1^, indicating that the absorption peaks of MAH-g-PLA showed enhanced carbonyl peak and ether bond compared with pristine PLA; the absorption peak of the C–H group appeared at 2926 cm^−1^, indicating that the hydrocarbon bond content of the alkane is increased. The above information indicated that MAH reacted with PLA during radical reaction in the presence of initiator DCP, and was successfully grafted to the macromolecular chain of PLA.

### 3.2. Grafting Degree of MAH-g-PLA

The grafting ratios of MAH-g-PLA are shown in Table 3, when the different addition amounts of MAH were added. As can be seen in Table 3, with the increase of MAH content, the grafting degree of MAH-g-PLA increased gradually. When MAH monomer content was 1.5 g, the grafting degree of MAH-g-PLA was the highest, and the grafting degree was 0.97%. The amount of MAH monomer was more than 1.5 g, the grafting degree of MAH-g-PLA was slightly reduced, which was because the MAH of high concentration inhibited the formation of grafting monomer. The MAH-g-PLA monomers with the highest grafting degree were used to prepare PLA/Wood fiber/MAH-g-PLA composites.

### 3.3. Performance of PLA/Wood fiber/MAH-g-PLA

#### 3.3.1. XRD and Crystallinity

**Figure 3:**
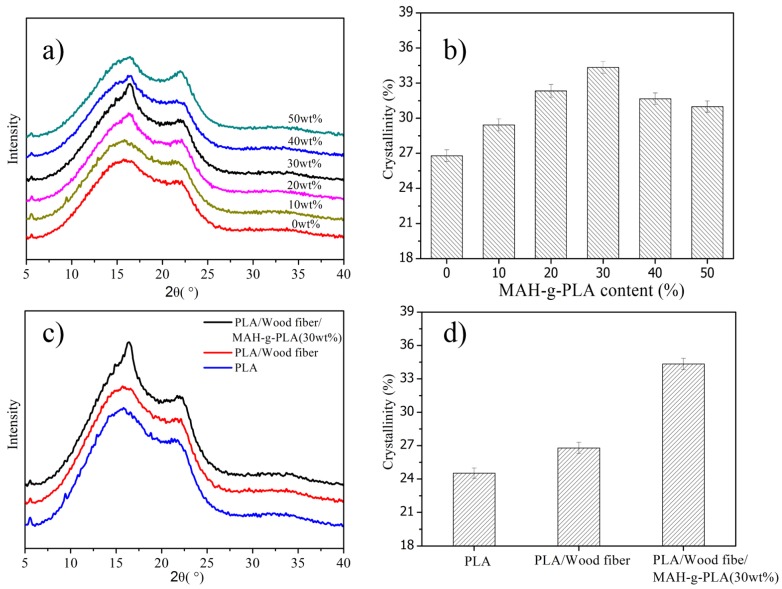
X-ray diffraction spectra and relative crystallinity graph of PLA/Wood fiber/MAH-g-PLA composites composite. (**a**,**c**) X-ray Diffraction Spectra; (**b**,**d**) Relative Crystallinity Graph.

Figure 3a,c showed the X-ray diffraction patterns of pristine PLA and PLA/Wood fiber/MAH-g-PLA composites with 0, 10, 20, 30, 40, or 50 wt % MAH-g-PLA, respectively, and the crystallinity of pristine PLA and the composites with different amounts of MAH-g-PLA were shown in Figure 3b,d. As can be seen in Figure 3a, the strength of the diffraction peaks increased with the addition of 10 to 30 wt % of MAH-g-PLA compared with no addition of MAH-g-PLA, showing the highest diffraction intensity at 30 wt% MAH-g-PLA addition, demonstrating that the addition of grafted PLA could improve the structural regularity of the composites, this was due to the increase in the content of β crystal in the composite [27,28]. Further increasing the content of MAH-g-PLA to 40 and 50 wt %, the intensity of the diffraction peak decreased gradually, which may be attributed to the extra MAH-g-PLA in the composites being increased in the entanglement density of the macromolecule chains, and the free movement between fibrous wood fiber molecules and PLA molecules being hindered. Therefore, increasing the molecular chain from the orderly disorder to disorder, the crystallinity of the polymer decreased [29,30]. Compared with pristine PLA, wood fiber can improve the diffraction intensity of composites, which can be seen in Figure 3c. In Figure 3b, when the amount of MAH-g-PLA was 10–50 wt %, the relative crystallinity of the composites increased by 10–28% due to the well blending of wood fiber and PLA under the compatibilization effect of MAH-g-PLA. The nucleation process of the crystallization reaction, the rearrangement of the PLA molecule, and the molecular crystal of the cellulose were accelerated, so that the fracture layer between two phases was thinner, the compatibility was better, so that the crystallinity of the composite was improved. Figure 3d showed the relative crystallinity of PLA was 24.52%, and the addition of wood fiber can promote the crystallization of polylactic acid. Adding 30 wt % of the MAH-g-PLA, the relative crystallinity of the composites increased to 34.34%, therefore, the addition of 30 wt % MAH-g-PLA showed the best reinforcement effect on the PLA/Wood composites.

#### 3.3.2. Mechanical Properties

**Figure 4:**
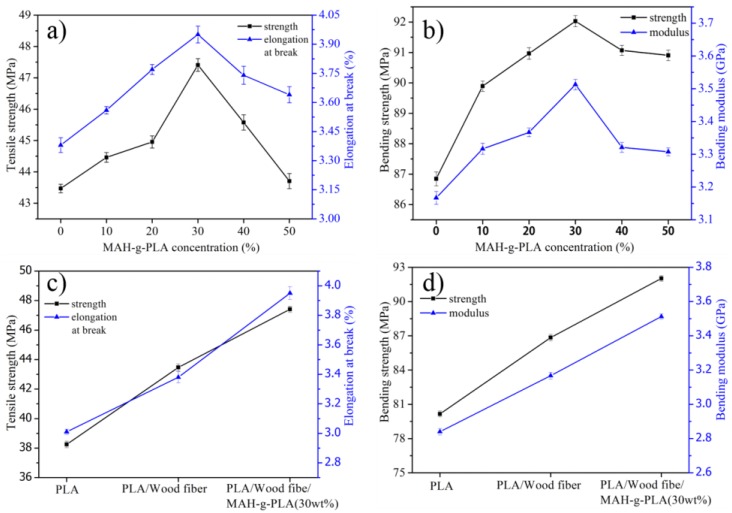
Mechanical properties of PLA/Wood fiber/MAH-g-PLA composites. (**a**,**c**) Tensile strength and elongation at break; (**b**,**d**) Bending strength and modulus.

The tensile strength and elongation at break of the composites are shown in Figure 4a,c, and the bending strength and modulus are shown in Figure 4b,d. From Figure 4a, it can be seen that the prepared MAH-g-PLA could improve the tensile strength and elongation at break of the composites. When adding 30 wt % of MAH-g-PLA, the tensile strength and elongation at break attained the highest value, 47.41MPa and 3.95%, respectively. The tensile strength of the composites modified by 30 wt % MAH-g-PLA was increased by 9% and the elongation at break increased by 17% compared to MAH-g-PLA-free composites, attributing that the MAH-g-PLA molecule displayed a similar structure to that of the PLA molecular chain and a structure similar to that of the MAH polar molecule [31]. The addition of the MAH-g-PLA made the PLA-wood fiber interface arrange more closely when external force was applied, leading to a fact that the stress could be easily transferred from PLA matrix to wood fiber. The transferring function between interface could, in turn, weaken the stress concentration in the composites, thereby increasing the tensile strength of the composites. The appropriate content of MAH-g-PLA compatibilizer can therefore create a better “coupling effect” in PLA/Wood fiber composites. The elongation at break of MAH-g-PLA modified composites were higher than those of unmodified composites. When the additional amount of MAH-g-PLA was more than 30 wt%, the elongation at break of the composites decreased gradually, which could be explained by the unreacted MAH groups in the system during twin-screw reaction extrusion reacting during further thermomechanical processing, increasing the crosslinking density between wood fibers and PLA matrix. From Figure 4c, it can be seen that wood fiber played a role in toughening polylactic acid. It can also be seen from Figure 4b that when the content of MAH-g-PLA was 30 wt %, the bending strength and modulus of the composites reached maximum values of 92.03 MPa and 3.51 GPa, respectively. The bending strength of the 30 wt % MAH-g-PLA modified composites was increased by 6% and the bending modulus increased by 11% compared to the composites without adding MAH-g-PLA. It can be discerned that the anhydride groups of MAH could react with the hydroxyl groups in PLA and wood fibers, which greatly improves the interfacial adhesion. The MAH-g-PLA therefore significantly improved the interfacial compatibility, resulting in an enhanced interfacial stability so that the molecular chains did not slide easily with each other. Moreover, the formation of a crosslinking network during the extrusion process could also lead to increased bending strength and modulus of the composites. When the amount of MAH-g-PLA was more than 30 wt %, the bending strength and modulus decreased gradually. This decrease of bending properties can be discerned that after the MAH-g-PLA reached saturation, the excess MAH-g-PLA stayed in the wood fiber and PLA, so that the molecular chains were easy to slide, resulting in a decrease in tensile strength and flexural modulus. As can be seen in Figure 4d, wood fiber can improve the anti-bending ability of PLA.

#### 3.3.3. Thermal Stability

**Figure 5:**
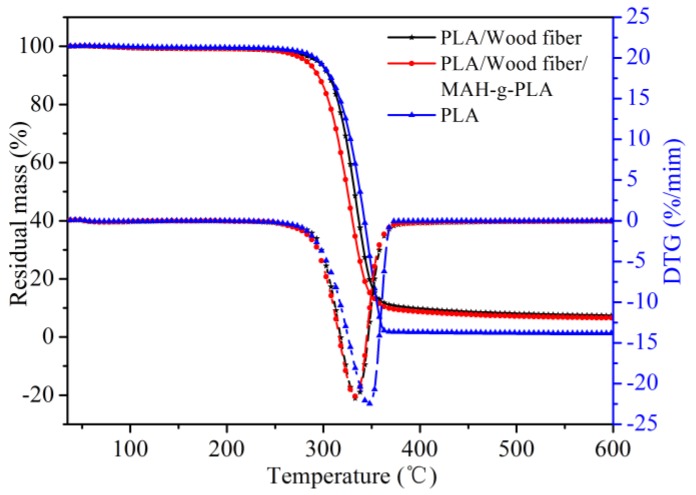
TGA and differential thermal gravity (DTG) curves of PLA, PLA/Wood fiber and PLA/Wood fiber/MAH-g-PLA composites.

The TGA and differential thermal gravity (DTG) curves of PLA, PLA/Wood fiber and PLA/Wood fiber/MAH-g-PLA are shown in Figure 5. It can be seen from Figure 5 that the thermal decomposition temperature of PLA/Wood was 276.3 °C and the thermal decomposition temperature of pristine PLA was 307.5 °C. Compared with pristine PLA, the initial decomposition temperature of composites decreased by about 30 °C, which was due to that the addition of wood fiber that could reduce the crystallinity of the PLA, and therefore, this resulted in a decrease in the molecular weight of the small amount of PLA during the molding process. Figure 5 also shows that the maximum decomposition rate of pristine PLA was greater than that of PLA/Wood composite, and that meanwhile, the maximum decomposition rate temperature of PLA/Wood fiber moved to lower temperature, which proved that the addition of wood fiber accelerated the thermal degradation of PLA. The MAH-g-PLA grafts were used as an interfacial compatibilizer to improve the compatibility of PLA molecular chains and wood fiber, when 30 wt % was added, as compared with no addition of MAH-g-PLA, the initial degradation temperature of the composites decreased slightly, indicating that the addition of MAH-g-PLA accelerated the initial degradation of the composites. This might be explained by the introduction of MAH molecules during the decomposition process, which could destroy the original crystal structure of PLA, promoting thermal decomposition of composites. At the same time, the maximum decomposition rate of the composites was decreased and the thermal decomposition residual of the composites increased when 30 wt % of the MAH-g-PLA was added.

#### 3.3.4. Dynamic Rhelogy

**Figure 6:**
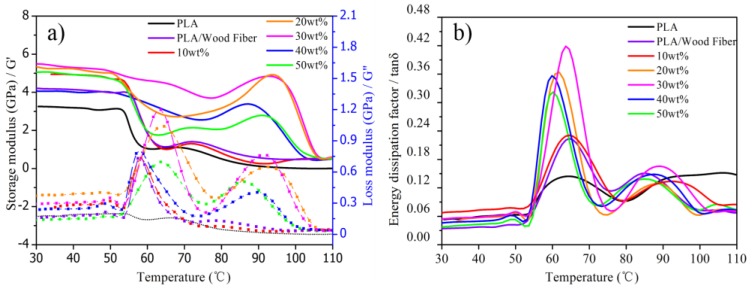
Dynamic mechanical thermal analysis (DMA) of PLA/Wood fiber/MAH-g-PLA composites. (**a**) Storage and Loss modulus curves; (**b**) Energy dissipation factor curves.

The curves of the storage modulus (G′) and the loss modulus (G′′) of PLA, PLA/Wood fiber, and PLA/Wood fiber/MAH-g-PLA in the range of 30 to 110 °C are shown in Figure 6a. Figure 6b shows the variation of the loss factor tanδ with temperature for the three composites. It can be seen from Figure 6a that the storage modulus of PLA/Wood fiber and PLA/Wood fiber/ MAH-g-PLA composites was significantly larger than that of pristine PLA in the temperature range of 30~110 °C. When 30 wt % of MAH-g-PLA was added, compared to pristine PLA and other additions content of MAH-g-PLA (10~20 wt %, 40~50 wt %), the composite material had the highest storage modulus. The temperature was at 30 °C, the storage modulus of PLA, PLA/Wood fiber, and PLA/Wood fiber/ MAH-g-PLA was 3.26 GPa, 4.06 GPa, and 5.48 GPa, respectively, indicating that the addition of wood fiber can improve the rigidity of PLA-based composites and improve the thermomechanical properties of the composites. Meanwhile, the loss modulus of PLA/Wood fiber/MAH-g-PLA composites was the largest among the all materials, indicating that the 30 wt % addition of MAH-g-PLA could lead to a more regular and stronger structure of composites, thereby improving the interfacial integrity of PLA and wood fiber composite to better transfer the stress under external force. When the temperature was above 75 °C, the storage modulus of the PLA/Wood fiber and PLA/Wood fiber/MAH-g-PLA composites showed a rising process, which may be due to the effect of the wood fibers, since there was a combination process of melting and recrystallization between the temperature and the molten state [32]. As shown in Figure 6a, it was shown that the addition of MAH-g-PLA can significantly improve the loss modulus of the composites, the effect of the 30 wt % addition of MAH-g-PLA was the best, indicating that MAH-g-PLA can improve the toughness of the composites, which can also be reflected in the increase in bending strength. It can be seen from Figure 6b that the glass transition temperature of PLA molecules in PLA/Wood fiber and PLA/Wood fiber/MAH-g-PLA composites was higher than that of pristine PLA, because wood fibers hinder the motion of the PLA molecular segment, thereby increasing the glass transition temperature of PLA molecules. When MAH-g-PLA was used as the interface compatibilizer, the glass transition temperature of the fiber molecules in the composite material moved to low temperature, demonstrating that the compatibility between the wood fiber and PLA was good.

#### 3.3.5. Water Absorption

**Figure 7:**
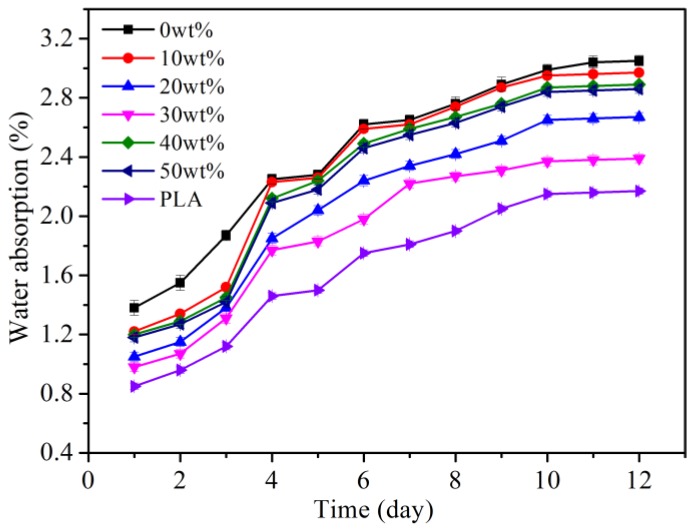
Water absorption of PLA/Wood fiber/MAH-g-PLA composites with different MAH-g-PLA additions.

The curves of the water absorption of PLA/Wood fiber/MAH-g-PLA composites with different MAH-g-PLA additions are shown in Figure 7. Since pristine PLA is hydrophobic, it had the lowest water absorption among these materials. Wood fiber is hydrophilic, the water absorption of PLA/Wood fiber composites therefore mainly depends on the water content of wood fiber, and the structural regularity of the composites [33]. It can be seen from Figure 7 that the water absorption of the composites increased gradually with time and eventually reached a saturation state. When no MAH-g-PLA was added, the water absorption of the composites was the highest, added with 10~50 wt %, the water absorption of the PLA/Wood fiber composites was significantly lower than that of the unmodified composites. This can be explained by the internal structure of the unmodified composites being loose, and that water molecules easily penetrated the composites. After the cross-linking of macromolecules in the composites by adding MAH-g-PLA, the structure of the composites turned more regular, so that water molecules did not so easily or deeply penetrate the composites. When the content of MAH-g-PLA was 30 wt %, the water absorption of the composites was the lowest, indicating that the interfacial compatibility of the composites was the best, which also reflected better crystallinity of the composites.

#### 3.3.6. Morphology Characterization

**Figure 8:**
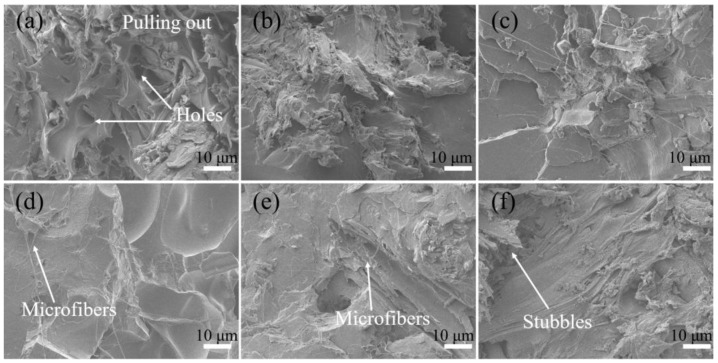
SEM images of composites with different contents of MAH-g-PLA: (**a**) 0 wt %; (**b**) 10 wt %; (**c**) 20 wt %; (**d**) 30 wt %; (**e**) 40 wt %; (**f**) 50 wt %.

The cross-sectional topography of PLA/Wood fiber/MAH-g-PLA composites with different contents of MAH-g-PLA are shown in Figure 8. The cross-sectional topography of PLA/Wood fiber without the interfacial compatibilizer is shown in Figure 8a. It can be observed from Figure 8a that there was an obvious phase separation between the wood fiber and PLA without adding the MAH-g-PLA, and the cross section was rough with grooves and holes. In fact, in the tensile test, some wood fibers could be extracted directly from the PLA matrix, indicating that the poor mechanical properties mainly resulted from the poor interfacial adhesion. In addition, the presence of gaps between the pores and the two phases indicated that without adding MAH-g-PLA, the motion of PLA molecules was restricted by poor interfacial compatibility, which did not completely fill the gap and pores between the wood fibers. It can be observed from Figure 8b–f that when 10–50 wt % of MAH-g-PLA was added, the voids and pores on the cross-section of the composites decreased, indicating that the surface became smoother and thicker in the process of hot pressing, and that the mobility of the PLA molecules was good, and that they could fill the gaps between wood fibers. At the same time, the uniform distribution of wood fiber in the PLA matrix retained strong toughness, allowing the material to bear a greater load in the tensile test. During tensile test, the wood fiber was not directly extracted from the PLA matrix after adding MAH-g-PLA, and wood fiber residues could be presented on the fractured surface, which could spread and transfer the stress, improving the mechanical properties. When the content of MAH-g-PLA was 30 wt %, the cross section of the composites and the compatibility of the wood fiber and PLA was the best, proving that the prepared MAH-g-PLA has a better compatibilizing effect on PLA/Wood fiber composites.

## 4. Conclusions

The MAH-g-PLA interfacial compatibilizer was prepared by melt extrusion in a twin-screw extruder, and the successful preparation was proved by infrared spectroscopy and chemical titration. The mechanical properties, thermal stability, dynamic thermodynamic properties, water absorption and interfacial compatibility of composites were studied by adding different amounts of MAH-g-PLA to PLA/Wood fiber composites. When MAH-g-PLA content was 30 wt %, the crystallinity of the composites was the highest, increased by 28%, as compared with no addition of MAH-g-PLA. The MAH-g-PLA acted as the interfacial compatibilizer, which can improve the tensile strength and flexural strength of the composites, and the addition of 30 wt % MAH-g-PLA shows the best results. The MAH-g-PLA can reduce the water absorption of the composites and the cross-sectional structure of the composites showed that the interfacial compatibility between the wood fiber and PLA phase significantly increased when 30 wt % of MAH-g-PLA was added.

## Figures and Tables

**Table 1:** The ratio of raw materials to the grafting reaction.

MAH (g)	DCP (g)	PLA (g)	Acetone (mL)
0.5	0.5	100	10
1.0	0.5	100	10
1.5	0.5	100	10
2.0	0.5	100	10
2.5	0.5	100	10

**Table 2:** The content of different raw materials in the composite.

Code	Wood Fiber (g)	PLA (g)	MAH-g-PLA (g)
1	60	240	0
2	60	216	24
3	60	192	48
4	60	168	72
5	60	144	96
6	60	120	120

**Table 3:** Grafting degree of the MAH-g-PLA with the different addition amounts of MAH.

MAH (g)	Grafting Degree (%)
0.5	0.45
1.0	0.51
1.5	0.97
2.0	0.91
2.5	0.86
